# Natural Killer Dendritic Cells Enhance Immune Responses Elicited by ****α****-Galactosylceramide-Stimulated Natural Killer T Cells

**DOI:** 10.1155/2013/460706

**Published:** 2013-06-26

**Authors:** Sung Won Lee, Hyun Jung Park, Nayoung Kim, Seokmann Hong

**Affiliations:** ^1^Department of Bioscience and Biotechnology, Institute of Bioscience, Sejong University, Seoul 143-747, Republic of Korea; ^2^Asan Institute for Life Sciences, Asan Medical Center, Seoul 138-736, Republic of Korea

## Abstract

Natural killer dendritic cells (NKDCs) possess potent anti-tumor activity, but the cellular effect of NKDC interactions with other innate immune cells is unclear. In this study, we demonstrate that the interaction of NKDCs and natural killer T (NKT) cells is required for the anti-tumor immune responses that are elicited by **α**-galactosylceramide (**α**-GC) in mice. The rapid and strong expression of interferon-**γ** by NKDCs after **α**-GC stimulation was dependent on NKT cells. Various NK and DC molecular markers and cytotoxic molecules were up-regulated following **α**-GC administration. This up-regulation could improve NKDC presentation of tumor antigens and increase cytotoxicity against tumor cells. NKDCs were required for the stimulation of DCs, NK cells, and NKT cells. The strong anti-tumor immune responses elicited by **α**-GC may be due to the down-regulation of regulatory T cells. Furthermore, the depletion of NKDCs dampened the tumor clearance mediated by **α**-GC-stimulated NKT cells *in vivo*. Taken together, these results indicate that complex interactions of innate immune cells might be required to achieve optimal anti-tumor immune responses during the early stages of tumorigenesis.

## 1. Introduction

The importance of communication among innate immune cells has recently been emphasized in various immunity models. For example, the interaction between dendritic cells (DCs) and natural killer (NK) cells influences both the innate and adaptive immune responses in anti-tumor and anti-microbial immunity [1]. DCs are specialized cells that engage in phagocytosis, antigen presentation, and the production of specific cytokines, including type I interferon (IFN) and interleukin (IL)-12, whereas NK cells can be cytolytic and secrete different cytokines, including IFN-*γ*, tumor necrosis factor (TNF)-*α*, and granulocyte-macrophage colony-stimulating factor (GM-CSF). Upon cellular interaction, NK cells are activated by the cytokines from DCs as well as by direct cell-cell contacts. Reciprocally, NK cells can induce DC maturation, which is important for optimal anti-tumor immunity. NK cells can also kill immature DCs. Thus, NK cells might regulate DC homeostasis. 

Natural killer T (NKT) cells also interact with NK cells and DCs. NKT cells are a group of heterogeneous T cells that share properties with both T cells and NK cells. NKT cells are characterized by the expression of an invariant T-cell receptor (TCR) encoded by V*α*14 and J*α*28 gene segments and V*β*8, 7, or 2 gene segments in mice. The cognate ligands of NKT cells are self and foreign glycolipids. The thymic development of NKT cells is dependent on CD1d, an MHC class I-like molecule. In addition to the swift secretion of cytokines, such as IFN-*γ* and TNF-*α*, NKT cells contribute significantly to regulating a variety of immune responses [2]. First of all, *α*-galactosylceramide-(*α*-GC-) activated NKT cells display potent anti-tumor activity. Moreover, *α*-GC-activated NKT cells quickly activate NK cells [3] and mature DCs, leading to adaptive immunity to tumor cells [4]. It has been shown that *α*-GC-pulsed DCs and NKT cells act cooperatively to enhance NK cytotoxicity [5]. NKT cells also interact with regulatory T cells (Tregs) [6–8]. Tregs, which express the Foxp3 transcription factor, play an essential role in maintaining self-tolerance by inhibiting effector T cells [9]. Several studies have demonstrated that Tregs generate a favorable environment for tumor growth by inhibiting anti-tumor effector T cells, such as CD8^+^ cytotoxic T cells [10, 11]. In patients with cancer, the number of Tregs increases in the peripheral blood as well as in the tumor microenvironment, and these Tregs may dampen anti-tumor immunity [12]. Thus, an intensive investigation has been performed to develop anticancer therapies that down-regulate Tregs.

A novel population of innate immune cells, which express both NK cell markers (NK1.1/DX5) and DC markers (CD11c), has been identified and named natural killer dendritic cells (NKDCs) [13] or IFN-producing killer dendritic cells (IKDCs) [14, 15]. The latter has been suggested to be a subset of NKDCs [16]. NKDCs present antigens using MHC class II molecules, similar to DCs, but NKDCs can also kill target cells with cytolytic molecules and produce IFN-*γ*, similar to NK cells [17]. Importantly, IKDCs have a more potent anti-tumor effect than NK cells, and have a broader range of target cells and prevent tumor outgrowth more effectively [15]. Nevertheless, the interaction of NKDCs with other innate immune cells has not yet been elucidated. This study was designed to investigate whether NKDCs participate in the anti-tumor immune responses triggered by *α*-GC using CD1d-deficient mice and V*α*14 TCR transgenic mice. The results suggest that *α*-GC-activated NKT cells interact with NKDCs, resulting in optimal anti-tumor responses as well as inhibition of Tregs.

## 2. Materials and Methods

### 2.1. Mice

C57BL/6 (B6) wild-type (WT) mice were purchased from Jung Ang Lab Animal Inc. (Seoul, Republic of Korea). CD1d knockout (KO) and V*α*14 TCR transgenic (Tg) mice were provided by Dr. A. Bendelac (University of Chicago, IL, USA). IL-12p35 KO mice were kindly provided by Dr. R. Locksley (University of California at San Francisco, CA, USA). CD11c-diphtheria toxin receptor (DTR) Tg mice were obtained from Dr. E. Choi (Seoul National University, Seoul, Republic of Korea). The mice used in this study were all from a B6 genetic background. The mice were bred and maintained at Sejong University and were 6–12-week old in the experiments performed, unless otherwise specified. The mice were fed a *γ*-irradiated sterile diet and autoclaved distilled water. The mice were injected intraperitoneally (i.p.) with vehicle or *α*-GC (2 *μ*g/mouse). The animal experiments were approved by the Institutional Animal Care and Use Committee at Sejong University (SJ-20100401009).

### 2.2. Cell Isolation and Culture

 For NKT cell isolation, whole splenocytes from V*α*14 TCR Tg mice were incubated with *α*-GC-loaded CD1d-dimer (PE-labeled; BD Biosciences, USA) at 37°C overnight. After washing with 1x PBS (phosphate-buffered saline), the cells were treated with PE-specific monoclonal antibody (mAb) for MACS (Miltenyi Biotec, Germany), following the manufacturer's instructions, and enriched for NKT cells by positive selection. The purity of the enriched cells was more than 95%. For the preparation of CD11c^+^ total DCs, whole splenocytes from WT, CD1d KO, or V*α*14 TCR Tg mice were stained with anti-CD11c mAb for MACS and enriched for CD11c^+^ DCs by positive selection. The DC population was >97% after MACS. To obtain the NK1.1^+^ DC and NK1.1^−^ DC populations separately, DCs were enriched from the spleens of either PBS- or *α*-GC-injected mice using CD11c MACS, stained with an anti-NK1.1 mAb, and then sorted using an FACSAria II cell sorter (BD Biosciences, San Jose, CA, USA). The NKDC population was >95% after sorting. B16 melanoma and YAC-1 tumor cell lines were cultured in RPMI 1640 (Gibco BRL, USA) culture media supplemented with 10% FBS, 10 mM HEPES, 2 mM L-glutamine, 100 units/mL penicillin-streptomycin, and 5 mM 2-mercaptoethanol.

### 2.3. Reverse Transcription Polymerase Chain Reaction (RT-PCR)

 Total RNAs were isolated from CD11c^+^ cells with Easy Red reagent (Intron, Republic of Korea) and were reverse transcribed into cDNA with oligo (dT) primers using M-MLV RT (Invitrogen life Technologies, USA) according to the manufacturers' instructions. The primers for *β*-actin were 5′-GTA TGG AAT CCT GTG GCA TC-3′ (forward) and 5′-AAG CAC TTG CGG TGC ACG AT-3′ (reverse). The primers for IFN-*γ* were 5′-AGC TCT TCC TCA TGG CTG TT-3′ (forward) and 5′-TTT GCC AGT TCC TCC AGA TA-3′ (reverse). The primers for TNF-*α* were 5′-GGC AGG TCT ACT TTG GAG TCA TTG-3′ (forward) and 5′-ACA TTC GAG GCT CCA GTG AAT TCG G-3′ (reverse). The primers for IL-12p40 were 5′-CAA GTG GGC ATG TGT TCC-3′ (forward) and 5′-TCT TCC TTA ATG TCT TCC AC-3′ (reverse). PCR was performed with a PCR Amplification Kit using 1 *μ*g of cDNA, 5 pmol of each primer, and HiPi PCR Premix (Elpis Biotech, Republic of Korea) in a 20 *μ*L reaction volume. PCR was performed in an XP Thermal Cycler (Bioer Technology, Hangzhou, China) with thermal cycling parameters as follows: 94°C for 5 min; 35 cycles of 94°C for 45 seconds, 57°C for 45 seconds, 72°C for 1 min; and 72°C for 5 min for IL-12p40, TNF-*α*, and IFN-*γ* and 94°C for 5 min 30 cycles of 94°C for 30 seconds, 54°C for 30 seconds, 72°C for 35 seconds; and 72°C for 5 min for *β*-actin. Equal amounts of RT-PCR product were run on a 1.5% agarose gel and stained with ethidium bromide. The intensity of the bands was measured with a Gel Logic100 image system (Kodak, NY, USA) and analyzed using Image J (National Institutes of Health software).

### 2.4. Flow Cytometry

 The following mAbs from BD Biosciences were used: fluorescein isothiocyanate (FITC)-, phycoerythrin (PE)-, or PE-Cy7-conjugated anti-TCR*β* (clone H57-597); allophycocyanin- (APC-) conjugated anti-CD25 (clone PC61); PE-conjugated anti-CCR5 (clone 2D7); PE- or APC-conjugated anti-CD4 (clone RM4-5); PE- or APC-conjugated anti-NK1.1 (clone PK-B6); PE-Cy7-conjugated anti-CD69 (clone H1.2.F3); APC-conjugated anti-CD3*ε* (clone 145-2C11); PE-conjugated anti-NKG2D (clone C7); PE-Cy7-conjugated anti-CD8 (clone 53-6.7); PE-conjugated anti-CD1d (clone 1B1); PE-conjugated anti-MHC II (clone M5/114.15.2); PE-conjugated anti-MHC I (clone KH95); biotin-conjugated anti-CD86 (clone GL1); PE-conjugated anti-CD40 (clone 3/23); PE-conjugated anti-FasL (MFL3); biotin-conjugated anti-TRAIL (clone N2B2); FITC-, PE-, or PE-Cy7-conjugated anti-CD11c (clone HL3); and PE-Cy7-conjugated anti-GITR mAb (clone DTA-1). The flow cytometry data were acquired with a FACSCalibur (Becton Dickinson, USA) and analyzed with the FlowJo software (Tree Star, USA). 

For surface staining, the cells were harvested and washed twice with cold 0.5% BSA-containing PBS (FACS buffer). To block the Fc receptors, the cells were incubated with anti-CD16/CD32 mAbs on ice for 10 min and were subsequently stained with fluorescence-labeled mAbs. For intracellular staining, splenocytes were incubated with Brefeldin A, an intracellular protein transport inhibitor (10 *μ*g/mL), in RPMI medium for 4 hours at 37°C. The cells were stained with FITC- or PE-conjugated anti-CD11c, PE-Cy7-conjugated anti-TCR*β*, and PE- or APC-conjugated anti-NK1.1 mAbs for 30 min at 4°C. The cells were then fixed with 1% formaldehyde in PBS and subsequently treated with permeabilizing solution containing 1% BSA and 0.1% saponin for 10 min at room temperature. Permeable cells were stained for an additional 30 min at room temperature with PE-conjugated anti-perforin, PE-conjugated anti-IFN-*γ*, PE-conjugated anti-IL-12p40, and FITC- or PE-conjugated isotype control rat IgG mAbs. More than 5,000 cells per sample were acquired with the FACSCalibur and analyzed with the FlowJo software.

### 2.5. Adoptive Transfer of DCs and *In Vivo* Tumor Metastasis Experiment

The total CD11c^+^-enriched DCs or NKDC-depleted DCs were prepared from the spleens of B6 WT mice using a MACS system with PE-conjugated anti-NK1.1 (clone PK-136) mAb, anti-PE microbeads, and anti-CD11c microbeads (Miltenyi Biotec). A total of 1.5 × 10^6^ DCs were transferred intravenously (i.v.) into CD11c-DTR recipient mice i.p. injected with 120 ng/mouse of diphtheria toxin (Sigma, USA) one day prior to the adoptive transfer of DCs. 

To establish the pulmonary metastasis model, CD11c-DTR Tg recipient mice were i.p. injected with DT (120 ng/mouse) to remove the CD11c^+^ DC population. After 16 hours, B16 melanoma cells (1 × 10^5^ cells/mouse) were i.v. transferred to the tail veins of DT-treated CD11c-DTR Tg mice simultaneously with either total DCs or NKDC-depleted DCs (1.5 × 10^6^ cells/mouse) prepared from WT mice. One hour later, the mice were injected i.p. with either vehicle or *α*-GC (2 *μ*g/mouse). Two weeks later, the mice were sacrificed, and subsequently, lung metastatic colonies were counted under a dissecting microscope.

### 2.6. Cytokine Assays

 The quantities of IFN-*γ* and TNF-*α* in culture supernatants were determined using sandwich ELISA according to the manufacturer's instructions (BD PharMingen, USA). The optical density at 450 nm was measured with an immunoreader (Bio-Tek ELX-800, USA).

### 2.7. Cytotoxicity Assay

 The flow cytometric CFSE/7-AAD cytotoxicity assay was performed as previously described [18] with minor modifications. DCs were isolated as described previously and suspended in RPMI medium. YAC-1 cells (3 × 10^6^) were labeled with 500 nM CFSE in Hanks' Balanced Salt Solution for 10 min at 37°C in a volume of 2 mL. The cells were washed twice in RPMI medium and used immediately. The CFSE-labeled target cells (20,000 cells) were incubated with DCs at different effector (E) : target (T) ratios (0 : 1, 3 : 1, 9 : 1, and 27 : 1). After 10 hours of incubation, the cells were stained with 0.25 *μ*g/mL of 7-AAD and were incubated for 10 min at 37°C in a CO_2_ incubator. The cells were washed twice with 1x PBS containing 1% FBS, designated as FACS buffer, and resuspended in FACS buffer. Cytotoxicity was assessed by flow cytometry.

### 2.8. Co-Culture and Transwell Assay in the Presence of Blocking Ab

Total CD11c^+^-enriched DCs or NKDC-depleted DCs were prepared from the spleens of B6 WT mice using MACS with PE-conjugated anti-NK1.1 (clone PK-136) mAb, anti-PE microbeads, and anti-CD11c microbeads (Miltenyi Biotec). Subsequently, these DCs were pulsed with 100 ng/mL of *α*-GC for 3 hours. After pulsing, the cells were washed with PBS to remove any excessive *α*-GC. The cells were co-cultured in the same wells as indicated for 72 hours. Alternatively, a transwell assay was performed using transwell plates (Costar, Corning Inc., NY, USA). NKT cells and DCs were placed in the lower chamber and the upper chamber of a 24-well plate, respectively, as indicated. After 72 hours of incubation, the NKT cells in the lower chamber were stained with PE-conjugated *α*-GC/CD1d-dimer, FITC-conjugated anti-CD3, and PE-Cy7-conjugated anti-CD69. Subsequently, the level of CD69 expression was examined on *α*-GC/CD1d-dimer^+^CD3^+^-gated NKT cells by flow cytometry. For blocking experiments using cytokine-specific mAbs, the same condition as above was used except for the addition of anti-IFN*γ* mAb (5 *μ*g/mL) to neutralize IFN-*γ*. IgG1 mAb (5 *μ*g/mL) was used as an isotype control. The purity of the NKT cells and CD11c^+^ DCs sorted by the MACS system was >95%.

### 2.9. *In Vitro* Treg Differentiation

Splenocytes from B6 and CD1d KO mice were isolated. Naive CD4^+^CD62L^+^ T cells were separated with a CD4^+^CD62L^+^ T cell isolation MACS kit (Miltenyi Biotec, Germany) according to the manufacturer's instructions. The isolated naive CD4^+^CD62L^+^ T cells (1 × 10^6^ cells/mL) were incubated in a 96-well plate precoated with anti-CD3*ε* (10 *μ*g/mL) and anti-CD28 (1 *μ*g/mL) mAb in 10% FBS RPMI media with hTGF*β* (10 ng/mL) and hIL-2 (100 U/well) for 5 days. DCs and NKDCs were added to assess their effect on the generation of Tregs. In some experiments, anti-IL-12 (5 *μ*g/mL; clone C17.8, BD Biosciences), anti-IFN-*γ* (5 *μ*g/mL; clone XMG1.2, BD Biosciences), and anti-TNF-*α* (5 *μ*g/mL; clone AF-410, R&D Systems) mAbs were added at the indicated concentrations.

### 2.10. Statistical Analysis

 Statistical significance was determined using Excel (Microsoft, USA). To compare two groups, Student's *t*-test was performed. *P < 0.05, **P < 0.01, ***P < 0.001 was considered significant.

## 3. Results

### 3.1. NKDCs Produce IFN-*γ* in the Presence of NKT Cells after *α*-GC Stimulation

**Figure 1:**
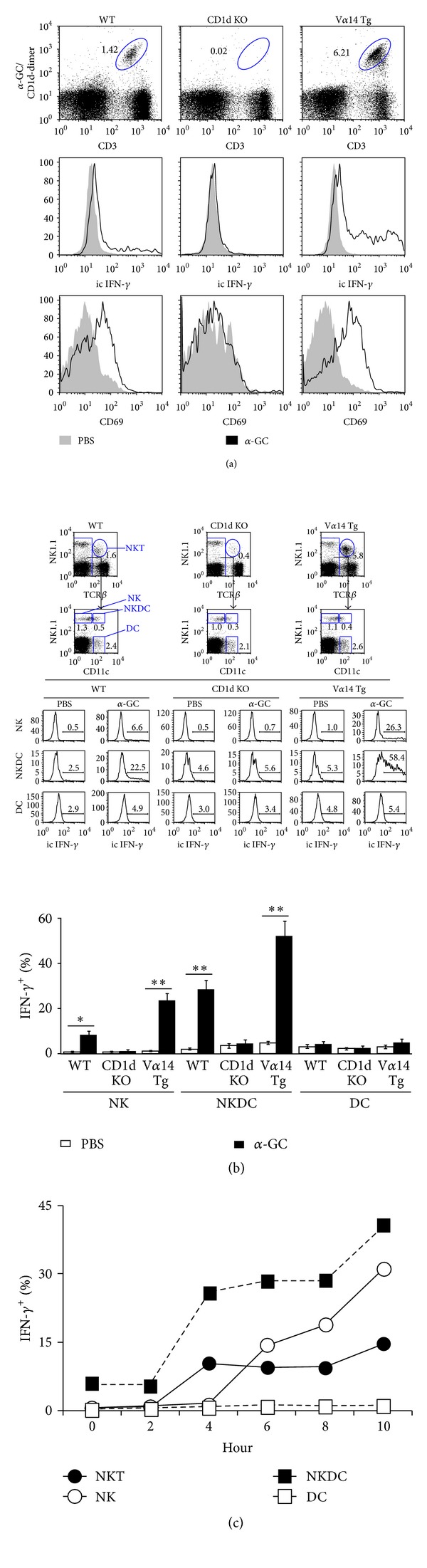
NKDCs produce IFN-*γ* in the presence of NKT cells following *α*-GC stimulation. (a-b) Splenocytes isolated from B6 WT, CD1d KO, and V*α*14 TCR Tg mice were stimulated with either PBS or *α*-GC (100 ng/mL) for 16 hours *in vitro*. (a) Intracellular IFN-*γ* production and surface CD69 expression were measured by flow cytometry in *α*-GC/CD1d dimer^+^-gated NKT cells (*n* = 3). (b) Intracellular IFN-*γ* production was assessed in NK cells (NK1.1^+^CD11c^−^TCR*β* ^−^), NKDCs (NK1.1^+^CD11c^+^TCR*β* ^−^), and DCs (NK1.1^−^CD11c^+^TCR*β* ^−^) by flow cytometry. Top, representative FACS plots; bottom, summary. The mean values ± SD (*n* = 3, *P < 0.05, **P < 0.01) are shown. Representative data from two independent experiments are shown. (c) Either *α*-GC (2 *μ*g) or PBS was i.p. injected into B6 WT mice. The intracellular expression of IFN-*γ* was measured in NK cells (NK1.1^+^CD11c^−^TCR*β* ^−^), NKT cells (NK1.1^+^CD11c^−^TCR*β* ^+^), NKDCs (NK1.1^+^CD11c^+^TCR*β* ^−^), and DCs (NK1.1^−^CD11c^+^TCR*β* ^−^) using flow cytometry at the indicated time points.

Although previous studies have demonstrated that *α*-GC-stimulated NKT cells induce the activation of DCs and NK cells, it was not clear whether activated NKT cells could stimulate NKDCs. To address this issue, we analyzed the NKDC population in wild-type (WT), CD1d-deficient (CD1d KO), and V*α*14 TCR transgenic (V*α*14 TCR Tg) mice. First, we confirmed the population and activity of NKT cells in the spleens of WT, CD1d KO, and V*α*14 TCR Tg mice as previously described (Figure 1(a)). As expected, CD1d KO mice have fewer NKT cells in the spleen, whereas V*α*14 TCR Tg mice have more NKT cells than WT mice. In addition, NKT cells from V*α*14 TCR Tg mice produced a large amount of IFN-*γ* and expressed high levels of the CD69 activation marker after *α*-GC stimulation* in vitro. *Intracellular IFN-*γ* in WT and V*α*14 TCR Tg NKT cells was increased significantly by *α*-GC, compared with vehicle control. 

To our surprise, the major producer of IFN-*γ* was NKDCs, rather than NK cells or NKT cells, and the production of IFN-*γ* by NKDCs was completely abolished in NKDCs from CD1d KO mice (Figure 1(b)). In contrast, a larger number of NKDCs from V*α*14 TCR Tg mice produced IFN-*γ* than those from WT mice. The cells analyzed in Figure 1(b) were incubated with *α*-GC at a single time point, 16 hours post-*α*-GC treatment. We assessed the kinetics of IFN-*γ* production to verify which cell type was the main producer of IFN-*γ* during the early innate immune response stimulated by *α*-GC *in vivo* (Figure 1(c)). Within 4 hours, a significant proportion of NKDCs began to produce IFN-*γ* and maintained the production of IFN-*γ* for more than 10 hours, as observed in Figures 1(b) and 1(c). The percentage of IFN-*γ*-producing NKDCs was higher than that of NK cells, NKT cells, and DCs without NKDCs at all time points. The kinetics of IFN-*γ* production was also assessed *in vitro.* NKDCs were the main producer of IFN-*γ* *in vitro* as well, but all the subsets appeared to start producing IFN-*γ* approximately 6 hours post-*α*-GC stimulation, slightly later than *in vivo*, and more NKT cells produced IFN-*γ* than NK cells (data not shown). In summary, these results suggest that the prompt activation of NKDCs by *α*-GC is dependent on NKT cells because *α*-GC itself lacks NKDC-stimulating adjuvant activity. 

### 3.2. *α*-GC Induces the Activation of NKDCs in the Presence of NKT Cells

**Figure 2:**
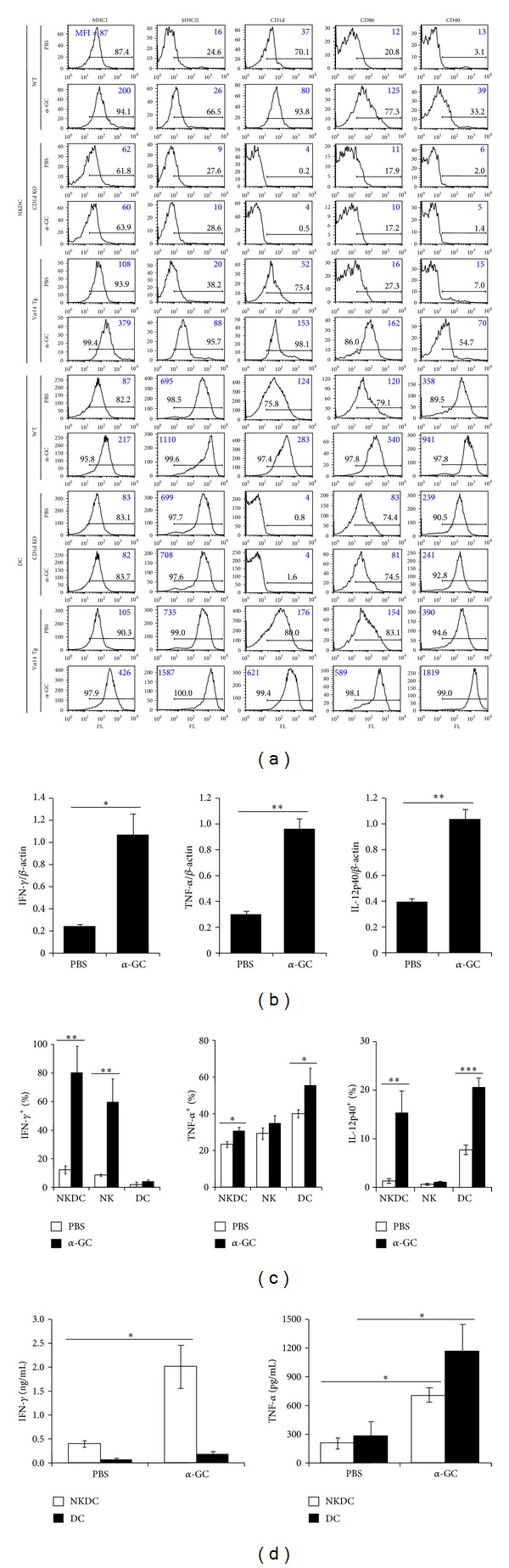
Alpha-GC induces the activation of NKDCs in the presence of NKT cells. (a) Either *α*-GC (2 *μ*g) or PBS was i.p. injected into WT, CD1d KO, and V*α*14 TCR Tg mice. Sixteen hours later, the surface expression of MHC class I, MHC class II, CD1d, CD86, and CD40 molecules on both NKDCs (NK1.1^+^CD11c^+^) and DCs (NK1.1^−^CD11c^+^) from isolated CD11c^+^ cells were assessed by flow cytometry. Representative data are shown (*n* = 5 for WT and CD1d KO mice; *n* = 2 for V*α*14 TCR Tg mice). (b) Total RNAs were isolated from CD11c^+^ cells. The transcripts of IFN-*γ*, TNF-*α*, IL-12p40, and *β*-actin were quantified using semiquantitative RT-PCR. These data are representative of three independent experiments. (c) Either *α*-GC (2 *μ*g) or PBS was i.p. injected into B6 mice. Sixteen hours later, the frequencies of IFN-*γ*-, TNF-*α*-, or IL-12p40-producing populations of splenic NKDCs (NK1.1^+^CD11c^+^TCR*β* ^−^), NK cells (NK1.1^+^CD11c^−^TCR*β* ^−^), and DCs (NK1.1^−^CD11c^+^TCR*β* ^−^) were assessed by flow cytometry. The mean values ± SD (*n* = 3, *P < 0.05, **P < 0.01, ***P < 0.001) are shown. (d) NKDCs and DCs from either PBS- or *α*-GC-treated mice were incubated with 100 ng/mL PMA for 20 hours in U-bottom 96-well plates (5 × 10^4^ cells/well). The levels of IFN-*γ* and TNF-*α* in the culture supernatant were measured using ELISA. The mean values ± SD (*n* = 3, *P < 0.05) are shown.

 It has been reported that stimulation with TLR ligands up-regulates MHC class II molecules and co-stimulatory molecules, such as CD40 and CD86, in NKDCs [14, 16]. To determine whether *α*-GC stimulation can induce the maturation of splenic NKDCs for antigen presentation, we examined the effect of *α*-GC stimulation on the expression of MHC and co-stimulatory molecules. We observed that *in vivo* *α*-GC administration up-regulated MHC class II, CD86, and CD40 expression in WT NKDCs (Figure 2(a)). These results are in line with a previous report in which a CpG oligomer was used as a stimulus [14]. CD1d KO mice did not show increased expression of these molecules in NKDCs. The number of NKDCs was also reduced in CD1d KO mice by approximately 15%, as compared with WT mice (data not shown). NKDCs and DCs from V*α*14 TCR Tg mice expressed more MHC class II and co-stimulatory molecules, as expected (percentages and mean fluorescence indices, MFI). These results suggest that NKDC maturation induced by *α*-GC requires NKT cells, implying that NKT cells play a necessary role in the development of NKDCs but not in the development of DCs.

Because *in vitro* *α*-GC stimulation resulted in IFN-*γ* production by NKDCs, the expression of pro-inflammatory cytokines was determined by detecting the transcription, intracellular production, and secretion in splenic NKDCs taken from WT mice injected with *α*-GC. First, the relative amounts of IFN-*γ*, TNF-*α*, and IL-12p40 transcripts were assessed by semiquantitative RT-PCR. *α*-GC stimulation *in vivo* up-regulated the transcripts encoding IFN-*γ*, TNF-*α*, and IL-12p40 in the CD11c^+^ DCs from WT mice in 16 hours (Figure 2(b)). We next examined whether the pro-inflammatory cytokine-producing NKDC populations were increased in response to *in vivo  *α**-GC treatment. The TNF-*α*-, IFN-*γ*-, and IL-12p40-producing cell populations were significantly increased in NKDCs from WT mice following *α*-GC stimulation (Figure 2(c)). To determine the amount of pro-inflammatory cytokines secreted by NKDCs, the NKDCs were sorted from the total CD11c^+^ DC population of *α*-GC-treated WT mice and subsequently stimulated with PMA. Twenty hours later, the cytokine levels in the culture supernatant were assessed using ELISA. As observed in Figure 2(d), NKDCs produce and secrete IFN-*γ* and TNF-*α* upon stimulation. *α*-GC-stimulated NKDCs secreted more IFN-*γ* but less TNF-*α* than DCs, in agreement with the intracellular staining observed in Figure 2(c). The results imply that NKDCs might have a specific role in inflammation. 

### 3.3. *α*-GC Enhances the Natural Cytotoxicity of NKDCs

**Figure 3:**
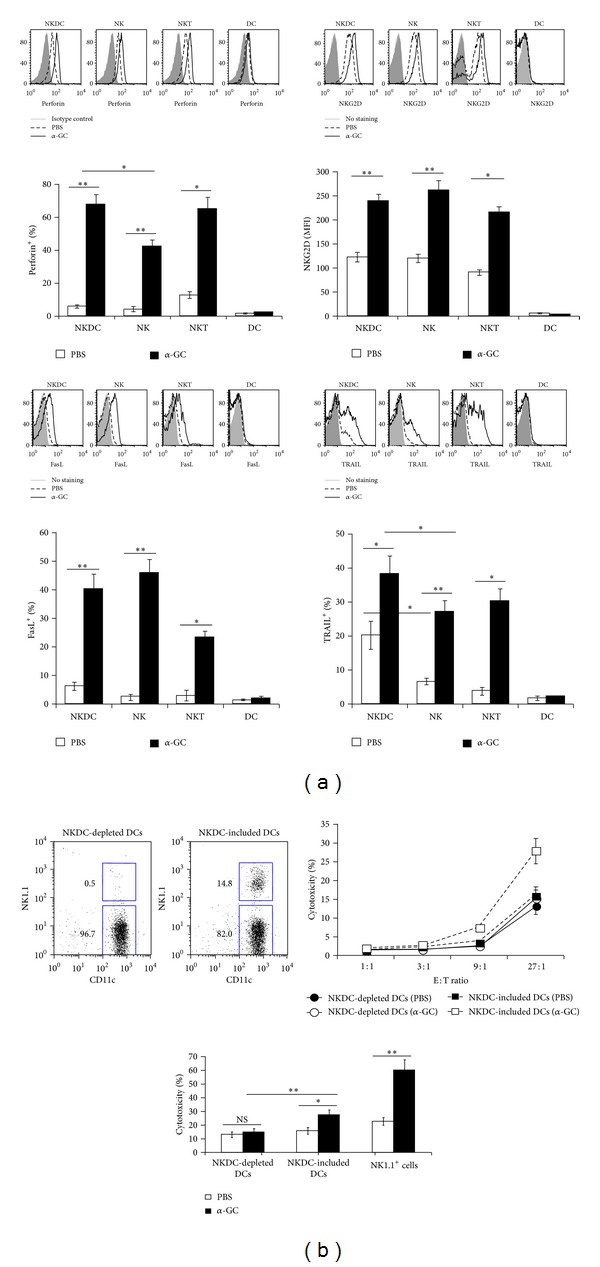
Cytotoxicity of NKDCs against tumor cells is enhanced by *α*-GC. (a) Either *α*-GC (2 *μ*g/mouse) or PBS was i.p. injected into B6 WT mice. Sixteen hours later, splenocytes were stained for flow cytometry. The expression of perforin (upper left panel), NKG2D (upper right panel), FasL (lower left panel), and TRAIL (lower right panel) was evaluated in NKDC (NK1.1^+^CD11c^+^TCR*β* ^−^), DC (NK1.1^−^CD11c^+^TCR*β* ^−^), NK (NK1.1^+^CD11c^−^TCR*β* ^−^), and NKT (NK1.1^+^TCR*β* ^+^CD11c^−^) populations. The mean values ± SD are shown (*n* = 3–6, *P < 0.05, **P < 0.01). (b) Either *α*-GC (2 *μ*g) or PBS was i.p. injected into B6 WT mice. Sixteen hours later, the total DCs or NKDC-depleted DCs isolated from these mice were used as effector cells for the cytotoxicity assay. The efficiency of depletion is shown in the upper panel. CFSE-labeled YAC-1 tumor cells were used as target cells. Effector cells were co-cultured with 2 × 10^4^ target cells at the indicated ratios. Cytotoxicity at the 27 : 1  E : T ratio is displayed in the lower panel. Cytotoxicity was evaluated by calculating the percentage of 7-AAD^+^ (dead) cells compared to CFSE^+^ target cells. The mean values ± SD are shown (*n* = 3).

Along with NK and NKT cells, NKDCs participate in tumor immunosurveillance by killing tumor cells [15]. The cytotoxicity of NK cells, NKT cells, and NKDCs is dependent on perforin, Fas ligand (FasL), and TNF-related apoptosis-inducing ligand (TRAIL). The expression of perforin, FasL, TRAIL, and NKG2D, an NK-cell-activating receptor expressed in all NK cell subsets, was assessed in NKDCs, NK cells, DCs, and NKT cells following *α*-GC treatment *in vivo *(Figure 3(a)). All of the cytotoxic molecules were produced at high levels in NKDCs in response to *α*-GC stimulation. Specifically, the frequency of perforin- and TRAIL-producing NKDCs was significantly higher than NK cells and comparable to NKT cells. However, the FasL-expressing NKDC population was comparable to or slightly smaller than the NK cell population and larger than the NKT cell population. The majority of NKDCs expressed NKG2D at similar levels to NK cells, and the mean fluorescence of NKG2D was enhanced by *α*-GC treatment. NKT cells were divided into NKG2D^+^ and NKG2D^−^ populations. *α*-GC treatment increased the NKG2D^+^ NKT cell population as well as the mean fluorescence. The differential expression of these molecules implies that the NK cells, NKT cells, and NKDCs might play different roles in anti-tumor immunity. In addition, the cytotoxicity of the total DC population was determined *in vitro*, as observed in Figure 3(b). The total *α*-GC-stimulated DC population killed YAC-1 tumor cells more efficiently than NKDC-depleted DC population. The cytotoxicity increased by approximately 200%. DCs did not produce detectable perforin, FasL, or TRAIL, and NKDC depletion from total DCs resulted in a dramatic decrease of *α*-GC-mediated cytotoxicity. These data strongly suggest that the NKDC population was responsible for the cytotoxicity of the total DC population. In conclusion, NKDCs work effectively for cytokine production (Figure 2) and cytotoxicity (Figure 3) in the presence of NKT cells upon *α*-GC stimulation.

### 3.4. NKDCs Are Required for the Optimal Immune Responses of NK Cells, NKT Cells, and DCs

**Figure 4:**
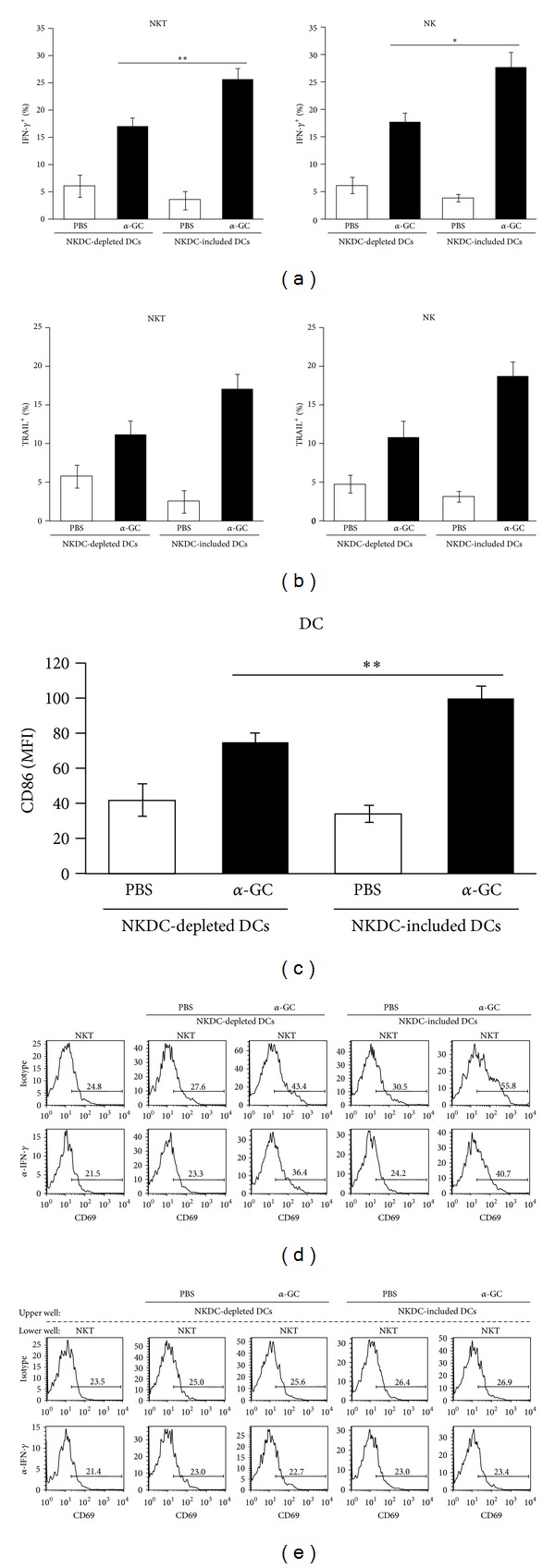
NKDCs are required for an optimal immune response in NK cells, NKT cells, and DCs. CD11c-DTR Tg recipient mice were i.p. injected with DT (120 ng/mouse) to remove the CD11c^+^ DC population. After one day, 1.5 × 10^6^ DCs (either total DCs or NKDC-depleted DCs) from WT mice were i.v. transferred to DC-deficient recipient mice. Eighteen hours later, splenocytes from both NKDC^+^ and NKDC^−^ mice were stained with mAbs for flow cytometry. (a) The percentage of the IFN-*γ*-producing population among NK1.1^+^CD3^−^CD11c^−^ NK cells and NK1.1^+^CD3^+^CD11c^−^ NKT cells was plotted. The mean values ± SD are shown (*n* = 3, *P < 0.05, **P < 0.01). (b) The percentage of the TRAIL-producing population among NK1.1^+^CD3^−^CD11c^−^ NK cells and NK1.1^+^CD3^+^CD11c^−^ NKT cells was plotted. The mean values ± SD are shown (*n* = 2). (c) The mean fluorescence of CD86 on CD11c^+^CD3^−^NK1.1^−^ DCs was plotted. The mean values ± SD are shown (*n* = 3, **P < 0.01). (d) NKT cells (6 × 10^5^ cells/well) were co-cultured with 2 × 10^5^ DCs (either total DCs (NKDC-included) or NKDC-depleted DCs pulsed with PBS or *α*-GC) in either the presence or the absence of anti-IFN-*γ* mAb. (e) For the transwell assay, 6 × 10^5^ NKT cells and 2 × 10^5^ DCs as described above were plated in a lower and an upper chamber, respectively, in either the presence or the absence of anti-IFN-*γ* mAb. After 72 hours of co-culture, CD69 expression of NKT cells was examined by flow cytometry. Representative results from two independent experiments are shown.

The communication between innate immune cells can be complex. In a previous report, *in vivo  *α**-GC injection rapidly activated NK cells to produce IFN-*γ* via the CD1d/NKT pathway [3]. In addition, in human umbilical cord blood samples, *α*-GC-pulsed DCs and NKT cells acted synergistically to enhance NK cytotoxicity *in vitro* [5]. NK cells require DCs to acquire full effector function *in vivo* in response to the bacterial-derived TLR ligand CpG [19]. Furthermore, the signaling pathways triggered by cell-cell interactions could be bi-directional, rather than uni-directional [20, 21]. Therefore, the effect of NKDCs on other innate immune cells, including NK cells, NKT cells, and DCs, was investigated. For this experiment, we took advantage of the CD11c-specific diphtheria toxin receptor (DTR)-expressing mice in which DCs can be depleted by a single i.p. injection of diphtheria toxin (DT). Twenty-four hours later, the total DCs or NKDC-depleted DCs from WT mice were prepared and were subsequently transferred i.v. to DC-depleted mice. *α*-GC was injected 1 hour later, and the immune cells were analyzed 18 hours after *α*-GC injection. Compared with mice with an intact DC population, substantially fewer NK cells and NKT cells from the NKDC-deficient mice produced IFN-*γ* and TRAIL (Figures 4(a) and 4(b)). In addition, CD86 expression was reduced on DCs from NKDC-deficient mice (Figure 4(c)). These results suggest that NKT cells promote NKDC activation, whereas NKDCs promote the function of NK cells, NKT cells, and DCs. 

The next question was whether the presence of *α*-GC-pulsed NKDCs could stimulate the NKT cells directly. To address this issue, co-culture and transwell assays were applied. Approximately 43% of NKT cells were activated when co-cultured with *α*-GC-pulsed DCs without NKDCs (NKDC-depleted DCs), whereas 56% of NKT cells were activated in the presence of *α*-GC-pulsed NKDCs (Figure 4(d)). However, the NKT cells did not become activated when they were separated from *α*-GC-pulsed DCs and NKDCs by a membrane in transwell plates (Figure 4(e)), indicating that direct interaction between NKT cells and DCs is crucial for NKT cell activation. In addition, blocking IFN-*γ* signaling with an anti-IFN-*γ* mAb partially decreased the expression of CD69 on NKT cells, which suggests that IFN-*γ* also plays a role in the activation of NKT cells by *α*-GC-pulsed NKDCs (Figures 4(d) and 4(e)). Thus, our results demonstrated that direct contact between NKT cells and NKDCs is required for NKT cell activation in addition to cytokines.

### 3.5. DC Subsets Additively Inhibit the Generation of Tregs after *α*-GC Stimulation *In Vitro*

**Figure 5:**
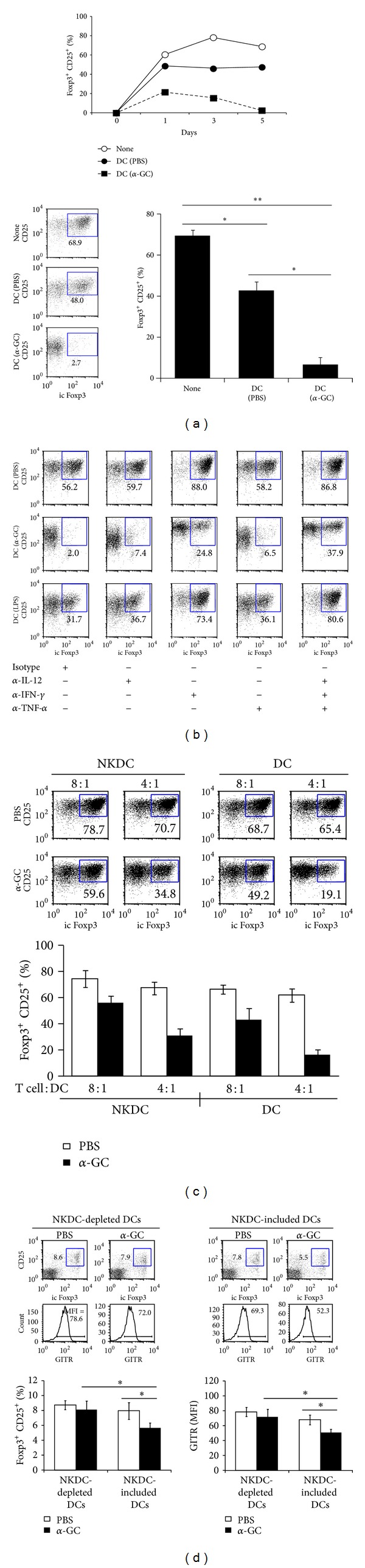
DC subsets additively inhibit the generation of Tregs after *α*-GC stimulation *in vitro. *(a) Naive CD4^+^CD62L^+^ T cells were isolated from B6 WT mice. These cells (1 × 10^5^ cells/well) were co-cultured in Treg-polarizing conditions with DCs (2 × 10^4^ cells/well) from either PBS- or *α*-GC-injected mice. In the upper panel, the percentage of iTreg cells among the total CD4^+^ T cells in culture was evaluated by flow cytometry at the indicated time points. In the lower panel, the frequencies of CD25^+^Foxp3^+^ iTreg cells on day 5 are shown (Mean values ± SD, *n* = 3, *P < 0.05, **P < 0.01). (b) Naive CD4^+^CD62L^+^ T cells were cultured for 5 days under Treg-polarizing conditions with DCs from either PBS- or *α*-GC-injected mice. LPS injection was performed as a positive control. Neutralizing mAbs specific to IFN-*γ* (5 *μ*g/mL), IL-12 (5 *μ*g/mL), or TNF-*α* (5 *μ*g/mL) were added during the culture. The percentage of CD25^+^Foxp3^+^ iTreg cells was evaluated by flow cytometry. These data are representative of two independent experiments. (c) Naive CD4^+^CD62L^+^ T cells were cultured for 5 days in Treg-polarizing conditions in the presence of either NK1.1^+^ DCs (NKDCs) or NK1.1^−^ conventional DCs (DCs) from either PBS- or *α*-GC-injected mice. The percentage of CD25^+^Foxp3^+^ iTreg cells was evaluated by flow cytometry. The mean values ± SD are shown (*n* = 2). (d) CD11c-DTR Tg recipient mice were i.p. injected with DT (120 ng/mouse) to remove the CD11c^+^ DC population. One day later, 1.5 × 10^6^ DCs (either total DCs or NKDC-depleted DCs) from WT mice were i.v. transferred to DT-treated CD11c-DTR Tg recipient mice. Subsequently, 1 hour later, *α*-GC was administered i.p. into both groups of mice. Three days later, splenocytes prepared from both groups were stained with mAbs for flow cytometry. The upper panels are dot plots of iTregs, and the middle panels are histograms of GITR expression on the iTreg population. The lower panels show the percentages of CD25^+^Foxp3^+^ iTreg population among CD3^+^CD4^+^ T cells. The mean values ± SD are shown (*n* = 3).

In a previous report, we demonstrated that induced regulatory T cell (iTreg) induction in CD1d KO mice was significantly increased *in vitro* and that the addition of NKT cells caused a remarkable reduction of iTregs, which was mediated by IFN-*γ* [8]. In this study, we investigated whether DC populations, including NKDCs, affected the function and differentiation of Treg cells. CD4^+^CD25^+^FoxP3^+^ iTreg populations were monitored during co-culture of naive CD4^+^CD62L^+^ T cells with total splenic DCs derived from vehicle- or *α*-GC-treated WT mice. It should be noted that the DCs used in this experiment were activated *in vivo* in the presence of NKT cells. As observed in Figure 5(a) (upper panel), iTregs were not efficiently induced by DCs from *α*-GC-treated mice, and iTreg numbers declined quickly compared with PBS-treated DCs or in the absence of DCs. On co-culture day 5, the iTreg population was only 2.7% when co-cultured with *α*-GC-treated DCs, whereas the iTreg populations were 68.9% and 48.0% when cultured without DCs and with PBS-treated DCs, respectively (Figure 5(a), lower panel). These results suggest a regulatory role for activated DCs in the differentiation of iTregs.

 The inhibitory effect of *α*-GC on Treg generation could be mediated by soluble factors produced by the DCs. DC-derived IL-12 may be involved in mediating the suppression of Treg cells because IL-12 plays a major part in promoting Th1 responses that inhibit Treg cell generation [22, 23]. IFN-*γ* and TNF-*α* were also observed to inhibit the differentiation and function of Treg cells [24, 25]. As *in vivo  *α**-GC injection strongly induced pro-inflammatory cytokines such as IFN-*γ*, TNF-*α*, and IL-12p40 (Figure 2(b)), the inhibitory effect of pro-inflammatory cytokines was tested in the DC-mediated suppression of iTreg differentiation using neutralizing antibodies specific to IFN-*γ*, TNF-*α*, and IL-12. Blocking IFN-*γ* restored iTreg generation most effectively, although blocking TNF-*α* or IL-12 partially restored iTreg generation. The neutralization of all 3 cytokines resulted in further restoration of Treg development to near normal levels (Figure 5(b)). In accordance with these results, the addition of IFN-*γ*, TNF-*α*, or IL-12 to co-cultured cells led to the down-regulation of Foxp3 expression in the CD4^+^CD25^+^ population (data not shown). These results suggest that the regulatory role of activated DCs and NKDCs in the differentiation of iTregs is mediated by multiple cytokines, including IFN-*γ*, TNF-*α*, and IL-12. 

The effect of NKDCs on iTreg generation was investigated. Naive CD4^+^CD62L^+^ T cells were co-cultured with NKDCs or conventional NK1.1^−^ DCs from *α*-GC-stimulated mice. Like DCs, *α*-GC-stimulated NKDCs reduced the percentage of Foxp3^+^ T cells in co-culture (Figure 5(c)), but the individual NKDC and NK1.1^−^ DC populations failed to reduce Foxp3^+^ T cells as dramatically as the total DC population (Figure 5(a)). These results suggest an additive regulatory effect of NKDC and NK1.1^−^ conventional DC populations on iTreg differentiation. 

To examine whether the inhibitory effect of NKDCs on Treg differentiation is also effective *in vivo*, we employed DT-treated CD11c-DTR Tg mice as DC-depleted recipients. Upon *in vivo  *α**-GC administration, mice that received an adoptive transfer with NKDC-depleted DCs displayed increased numbers of Treg populations and enhanced levels of glucocorticoid-induced TNFR-related (GITR) expression on Treg cells, in comparison with mice receiving a transfer with the whole CD11c^+^ DC population (Figure 5(d)). These results suggest that NKDCs contribute to the suppression of Treg functions in *α*-GC-mediated *in vivo* responses.

### 3.6. NKDCs Reduce Tumor Metastasis in the Presence of *α*-GC *In Vivo*

**Figure 6:**
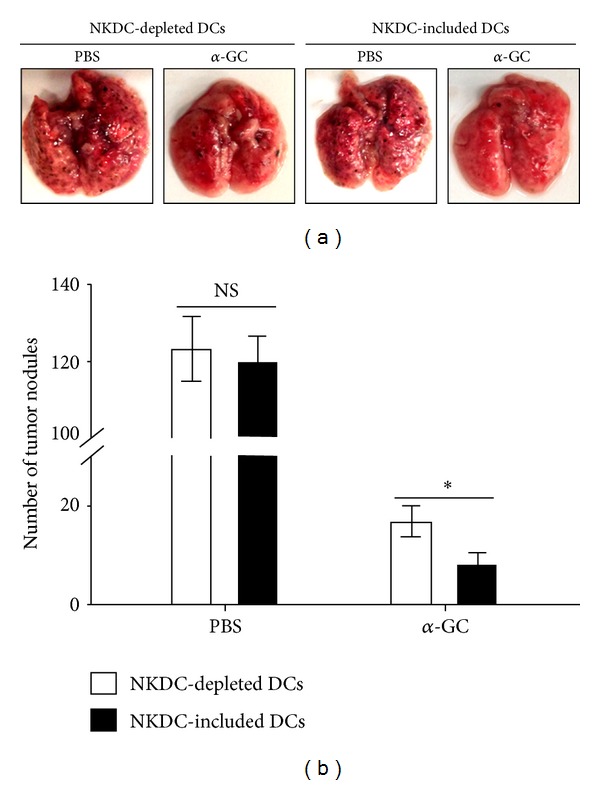
NKDCs mediate the anti-metastatic effect following *α*-GC stimulation. B16 tumor cells (1 × 10^5^/mouse) and 1.5 × 10^6^ DCs (either total DCs or NKDC-depleted DCs) from WT mice were i.v. transferred to DT-treated CD11c-DTR Tg mice. One hour later, *α*-GC was administered i.p. into both groups of mice. On day 14 after injection, mice were sacrificed, and the number of metastatic tumor nodules in the lung was counted. (a) Representative photos of lung samples from each group are shown. (b) The mean numbers of metastatic melanomas in the lung are shown. Representative results from two independent experiments are shown. The mean values ± SD are shown (*n* = 3 for each experiment, *P < 0.05).

As seen in Figure 3 and previously described, NKDCs could kill tumor cells quite efficiently. Thus, an *in vivo* experiment was set up to investigate the effect of NKDCs on cancer metastasis in the presence of *α*-GC, using CD11c-DTR Tg mice and a B16 melanoma lung metastasis model. In brief, NKDC-depleted DCs and total DCs (NKDC-included DCs) were adoptively transferred to DT-treated CD11c-DTR Tg mice at the same time of tumor cell injection i.v., and 1 hour later, *α*-GC was injected. NKDCs reduced the lung metastasis of B16 melanoma when immediately followed by *α*-GC-stimulation (Figure 6). The reduction in the numbers of tumor nodules by *α*-GC-pulsed NKDCs was significant compared with vehicle controls (PBS) as well compared with DCs without NKDCs. These results strongly suggest that NKDCs promote *α*-GC-elicited anti-tumor immunity *in vivo*.

## 4. Discussion

The phenotype and function of NKDCs have been thoroughly investigated in recent years, but cell-cell interaction of NKDCs with other innate immune cells has not been shown previously. Herein, we demonstrated that the interaction between NKDCs and NKT cells was required for optimal anti-tumor immunity mediated by *α*-GC. NKDCs were a major source of IFN-*γ* in the early hours after *α*-GC administration, and IFN-*γ* production depended on the presence of NKT cells. An increased amount of IFN-*γ* was detected by measuring RNA, intracellular protein, and secreted protein levels. Although qPCR was performed with total DCs to assess IFN-*γ* transcripts, based on the data with intracellular staining and ELISA, it was obvious that the main producer of IFN-*γ* was NKDCs, not DCs without NKDCs. NKDCs expressed an increased level of various DC and NK cell markers in the presence of NKT cells following *α*-GC stimulation. The expression levels of MHC class II and CD40 molecules in NKDCs were lower than DCs, implying that NKDCs may be less effective than conventional DCs at presenting antigens. It should be mentioned that NKDCs do express CD1d; therefore, we do not exclude a possible direct role for CD1d on the function and maturation of NKDCs. However, NKDCs from V*α*14 TCR Tg mice presented increased IFN-*γ* production compared with WT mice, clearly indicating that NKT cells are required for NKDC function. NKDCs also expressed high levels of cytotoxic molecules, and unsurprisingly, NKDCs killed tumor cells effectively, as shown previously. It was particularly interesting that NKG2D expression was up-regulated in NKDCs by *α*-GC. NKG2D is a well-studied NK cell-activating receptor, whose ligands are retinoic acid early inducible gene 1 (RAE-1), H60, murine UL-16 binding protein-like transcript 1 (MULT-1) in mice, and MHC class I chain-related molecules (MICA and MICB) and UL-16 binding proteins (ULBP-1, 2, and 3) in humans. MICA/B up-regulation is found in many cancer tissues, including hepatocellular carcinoma, rectal cancer, and osteosarcoma [26–28]. Hence, the up-regulation of NKG2D could contribute to the efficient killing of tumor cells.

 Not only is *α*-GC stimulation of NKT cells required for NKDC function, but also NKDCs are required for optimal NKT function, which was demonstrated by the adoptive transfer of conventional DCs without NKDCs. Finally, we observed that the down-regulation of Tregs might explain the enhanced anti-tumor immunity stimulated by *α*-GC. The down-regulation of Tregs was additively mediated by conventional DCs and NKDCs expressing IL-12, IFN-*γ*, and TNF-*α*. A previous study indicated that tumor cells can induce Tregs via a TGF-*β*-dependent pathway, leading to inhibition of anti-tumor immunity by Tregs [29]. It has also been reported that Tregs can inhibit tumor-infiltrating DCs [30]. Upon stimulation with *α*-GC, the pro-inflammatory cytokines secreted by DCs might disrupt the expression of Foxp3 and consequently impair Treg function [31]. Optimal anti-tumor immune responses might be achieved due to immunostimulatory DCs overriding Treg function. In this study, we demonstrated that NKDCs, functioning as immunostimulatory DCs, could be a primary mediator of the *α*-GC-mediated immune response. Thus, the modulation of Treg function via NKDC-NKT cell interactions could play a vital role in shifting the response toward Th1 types of immune responses, which are favorable to anti-tumor immunity [32]. In addition, we observed that GITR expression on iTregs was down-regulated by *α*-GC. Because it has been reported that GITR engagement enhances the proliferation of Tregs both *in vitro* and *in vivo* [33], down-regulation of GITR on Treg cells through NKDCs upon *α*-GC stimulation could result in better anti-tumor immune responses. Interestingly, GITR-expressing Tregs are increased in tumor-positive lymph nodes from advanced breast cancer patients [34].

In our previous study, the activation of NKT cells by *α*-GC, a specific NKT cell agonist, resulted in the inhibition of the development of iTreg [8]. There are other papers suggesting an interaction between NKT cells and Tregs. Intriguingly, Hua et al. suggested that liver NKT cells promoted Tregs and that Tregs inhibited NKT cells in CD1d-deficient mice [6]. However, they analyzed the immune cells after 3 days, whereas we analyzed them after 16 hours. It should also be noted that the liver is an immune-privileged organ with distinct immune cell populations. Hongo et al. suggested that the interactions between NKT cells and Tregs are required to tolerate combined bone marrow and cardiac transplant mice [7], though their earliest analysis time point for the immune cell population was 48 hours. In addition, graft rejection is biologically dissimilar to anti-tumor immunity. We speculate that strong, early innate immune responses could result in feedback responses, in an attempt to restore homeostasis. The feedback control of Treg homeostasis by DCs has been shown previously *in vivo* [35]. Alternatively, there might be a molecular or cellular switch for anti-tumor immune responses or tolerance. It has been shown that TLR signaling can determine the outcome of DC maturation as pro-inflammatory or tolerogenic after CD1d-mediated interaction with NKT cells [36]. Overall, these data suggest that there must be a fine-tuning of the innate immune response and that the perceived inconsistency between the two previously published papers and ours might highlight the complexity of these interactions.

IFN-*γ* plays a role in the activation of NKT cells by *α*-GC-pulsed NKDCs, but the effect was only partial. As it has been reported that NKDCs secrete IFN-*γ* via IL-12's autocrine effects in response to CpG administration [13], the following question emerged: is the production of IFN-*γ* by NKDCs upon *α*-GC stimulation dependent on IL-12 secretion? To address this issue, we assessed pro-inflammatory cytokine production by NKDCs after *α*-GC stimulation using IL-12p35 KO mice lacking functional IL-12. No significant differences in the intracellular IFN-*γ* levels were detected between the NKDCs of WT and IL-12p35 KO mice (data not shown), suggesting that NKDC activation by *α*-GC stimulation is not dependent on the IL-12 pathway. Taken together, we hypothesize the sequential interactions of NKT cells, NKDCs and Tregs are as follows: NKDCs express CD1d molecules at their surfaces, presenting glycolipid antigens, such as *α*-GC, to NKT cells. Swift activation of NKDCs following *α*-GC-mediated activation of NKT cells suggests that NKDCs might respond directly to cytokines that are immediately released by activated NKT cells. Alpha-GC-activated NKDCs not only produce the pro-inflammatory cytokines but also reduce the generation and function of Tregs via cytokines. Consequently, NKDCs can produce perforin, FasL, and TRAIL to enhance anti-tumor effects.

## 5. Conclusions

In conclusion, our findings in this study provide evidence for the first time that NKDCs are one of the main mediators of immune responses triggered by *α*-GC stimulation via boosting NKT cells and inhibiting Treg generation. The absence of NKDCs during the early anti-tumor immune response elicited by *α*-GC stimulation dramatically reduced the activation of other innate immune cells, including DCs, NK, and NKT cells, which strongly suggests that early anti-tumor immunity requires specific interactions among NKT cells, NKDCs, DCs, and Tregs after *α*-GC stimulation. However, it is still possible that other cell types other than the aforementioned cells are involved in this immune response. For example, *γδ* T cells amplify the innate and adaptive responses to *α*-GC [37]. A complex interaction of innate immune cells is required for the fine-tuning of optimal anti-tumor immunity in the early stages of tumorigenesis. The detailed molecular mechanisms of this phenomenon await further study, which may lead to novel anti-cancer immune therapies.
